# Gas Sensing Properties of In_2_O_3_ Nano-Films Obtained by Low Temperature Pulsed Electron Deposition Technique on Alumina Substrates

**DOI:** 10.3390/s18124410

**Published:** 2018-12-13

**Authors:** Tommaso Addabbo, Mara Bruzzi, Ada Fort, Marco Mugnaini, Valerio Vignoli

**Affiliations:** 1 Department of Information Engineering and Mathematical Science, University of Siena, via Roma 56, 53100 Siena, Italy; addabbo@dii.unisi.it (T.A.); marco.mugnaini@unisi.it (M.M.); valerio.vignoli@unisi.it (V.V.); 2 Department of Physics and Astronomy, University of Firenze, via G. Sansone, 1, 50019 Sesto Fiorentino (FI), Italy; mara.bruzzi@unifi.it

**Keywords:** metal oxide gas sensors, nano-structured device, pulsed electron deposition technique, sensor modeling

## Abstract

Nanostructured Indium(III) oxide (In_2_O_3_) films deposited by low temperature pulsed electron deposition (LPED) technique on customized alumina printed circuit boards have been manufactured and characterized as gas sensing devices. Their electrical properties have monitored directly during deposition to optimize their sensing performance. Experimental results with oxidizing (NO_2_) as well as reducing (CO) gases in both air and inert gas carriers are discussed and modeled.

## 1. Introduction

In recent years, Indium(III) oxide (In_2_O_3_) has shown a potential as a promising new material for gas sensing applications, in particular suitable for the detection of low concentrations of oxidizing gases like O_3_, NO_x_, and Cl_2_ [1,2,3,4]. On the other hand, it was also shown that In_2_O_3_-based gas sensors may have sufficient sensitivity and good selectivity toward some reducing gases (such as CO or H_2_) [5,6], this latter property strongly depending on the preparation route and surface stoichiometry. In fact, it is known that sensing performance of a metal oxides sensor is mainly regulated by its microscopic morphology and defect chemistry, material properties largely depending on the manufacturing process. In this regard, performance of gas sensors has been shown to be really boosted by using nano-structured materials [1,2,3,6].

In past, In_2_O_3_ nano-structured films have been manufactured by many different techniques such as chemical vapor deposition, thermal oxidation of In films, spray pyrolysis, sol–gel, atomic layer deposition, pulsed laser ablation, DC, and RF-sputtering [7]. These techniques generally require a complex processing for precursors to be formed in the environment of the deposition chamber. Furthermore, they require rather high temperatures of the substrate, and therefore generally do not allow using substrates already equipped with screen printed circuitry, as in the case of gas sensing devices, which will be damaged or even melted by high temperatures. 

In particular, papers by Korotcenkov et al. (e.g., [8,9]), studied deeply the behavior of In_2_O_3_ sensors grown by spray pyrolysis, discussing thoroughly the influence of the sensor microstructure on their performance.

In this work, we deposited nano-crystalline layers of In_2_O_3_ on dedicated printed circuit boards (PCBs). We used a novel deposition method, the low temperature pulsed electron deposition (LPED) technique, where a pulsed high power electron beam, permitting the non-equilibrium extraction of energetic particles, allows for depositing homogeneous films with the proper stoichiometry of almost any kind of materials at ambient temperatures, so that direct deposition on temperature-sensitive substrates as PCBs is made possible. In particular, for the sensors presented in this paper the LPED technique was applied with the simultaneous measurement of the film resistance during the growth, which is a specific and novel characteristic of our deposition procedure.

Based on previous works from Korotcenkov, we analyzed and modeled the behavior of our In_2_O_3_ nano-films deposited by LPED as NO_x_ and CO sensors. Two models are compared to discuss the behavior of our sensors, one where a ‘dense’ thin film is taken into account, and the other where ‘porous’ films are considered, this latter as already modeled in [10]. The comparison led to the conclusion that, for film thicknesses which bring to reasonable values for the film resistance, the ‘dense’ thin film model is the one that best fits the experimental results, which are in agreement with the ones reported by Korotcenkov in [9]. In particular, reducing the film thickness and the grain size decreases the time constants of the gas response of In_2_O_3_-based gas sensors, and increases the response to oxidizing gases (thanks also to an increased gas permeability of the film).

## 2. Sensor Preparation and Characterization

### 2.1. Thin Film Deposition and Characterization

**Figure 1:**
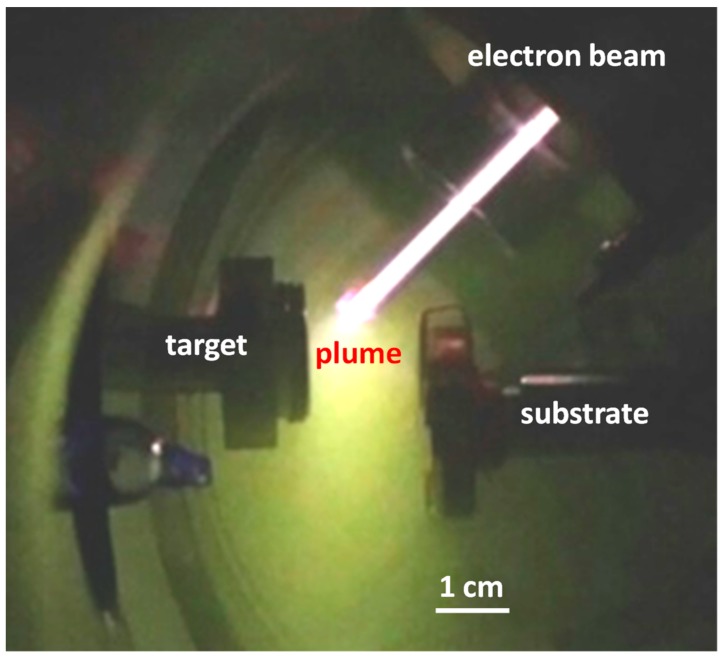
Photograph of the system during deposition of the In_2_O_3_ film; gas used is oxygen.

Thin films were deposited by low temperature pulsed electron deposition (LPED). This deposition system [11,12,13] has been developed by Organic Spintronics, Bologna, Italy [14]. LPED is obtained by means of a pulsed (100 ns), high power (500 MW/cm^2^), electron beam that penetrates approximately 1 µm into the target resulting in a rapid evaporation of target material, and its transformation in plasma state. Material of the ablation cloud is used for deposition on the substrate. The non-equilibrium extraction of the target material facilitates stoichiometric composition of the plasma. The formation of a dense discharge plasma increases the energy of the ablated particles to several tens of eVs, higher than during electron beam evaporation or sputtering. This makes it possible to deposit a dense and almost homogeneous nano-crystalline film even at room temperature. In this way, growing of adhering dense layers is made possible even on temperature-sensitive substrates with high energy conversion efficiency. The LPED accelerates electrons using a hollow cathode connected to a dielectric capillary [11,12,13,14]. High voltage is obtained by limiting the lateral expansion of charge carriers by means of a thin dielectric tube for channel confinement, in such a way that breakdown voltage is governed by the channel diameter while the length of the channel plays a minor role [15]. A transient hollow cathode (THC) is connected to the dielectric acceleration tube, filled with gas at low pressure. High voltage is applied to the THC. A photograph of the deposition chamber during growth of the In_2_O_3_ film is shown in Figure 1; gas used is oxygen. Typical operational characteristics of the system are a beam power density of about 500 MW/cm^2^, repetition rate around 20 Hz, energy efficiency about 30%, growth rate about 0.01 nm/s.

**Figure 2:**
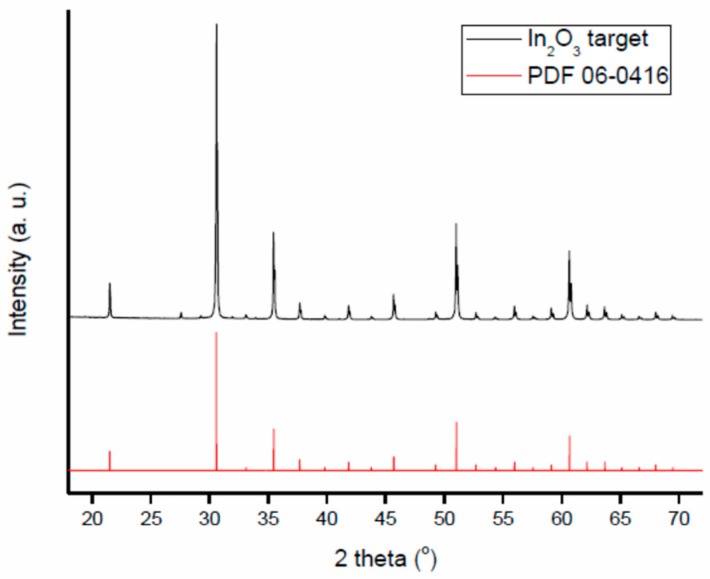
XRD analysis of the target as compared with the reference pattern PDF 06-0416.

Target used to deposit the In_2_O_3_ films studied in this work is a commercial indium oxide pellet (Testbourne Ltd., Basingstoke, United Kingdom, I1-9002-D3). The X-ray diffraction pattern of In_2_O_3_ target material is shown in Figure 2 and compared to reference pattern (PDF 06-0416). The pattern has been measured with angles 2θ ranging from 18° to 75°. Data, obtained using a D8 Advance Brucker powder diffractometer, CuKα1 radiation, show a low level of impurities.

**Figure 3:**
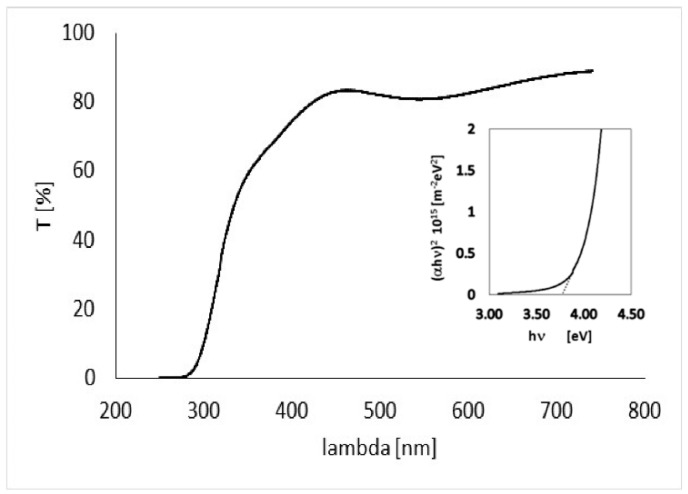
Optical transmission of a In_2_O_3_ 230 nm-thick film on glass substrate.

The quality of the deposited film, prior to preparing gas sensing devices, has been accurately analyzed on dedicated test samples. In_2_O_3_ thin films with thickness around 230 nm have been deposited on glass windows, and have been studied by means of optical transmission measurements, SEM analysis, microanalysis, and profilometry. Optical transmission measurements as a function of wavelength obtained with our film placed normally to the beam in a PerkinElmer spectrophotometer are shown in Figure 3.

From data shown in Figure 3, the absorption coefficient of the film as a function of the wavelength can be evaluated

(1) $$\alpha =\frac{1}{d}\;ln\left(\frac{100}{T\left(\%\right)}\right)$$

where *d* = 230 nm is the thickness of the deposited film. For direct optically-allowed inter-band transitions, the following relationship holds [16] $\alpha \propto \frac{1}{\nu }\sqrt{h\nu -E_{g}}$. So, energy gap *E_g_* can be then estimated by fitting with a linear curve the function (*αh υ*)^2^ vs. *hυ* (see plot in inset), where *υ* is the wave frequency and *h* the Planck constant. The energy gap so obtained, *E_g_* = 3.7 eV, is in agreement with what expected for pure In_2_O_3_ [17].

**Figure 4:**
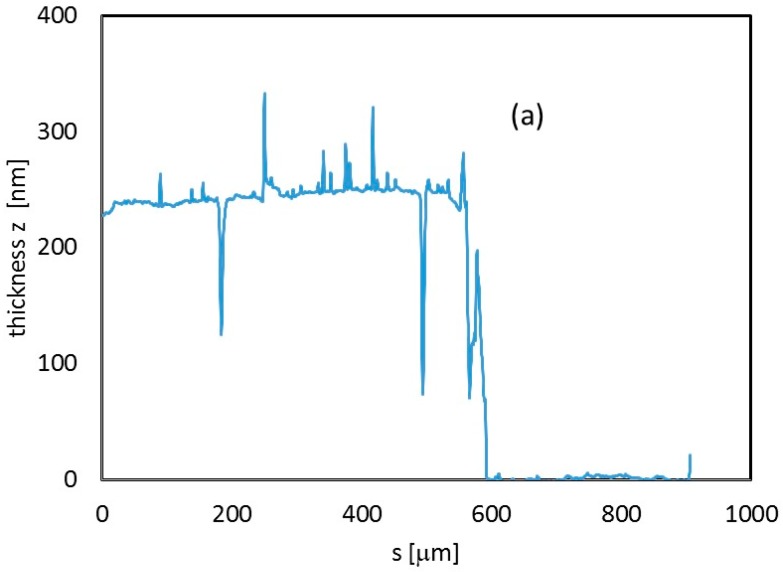

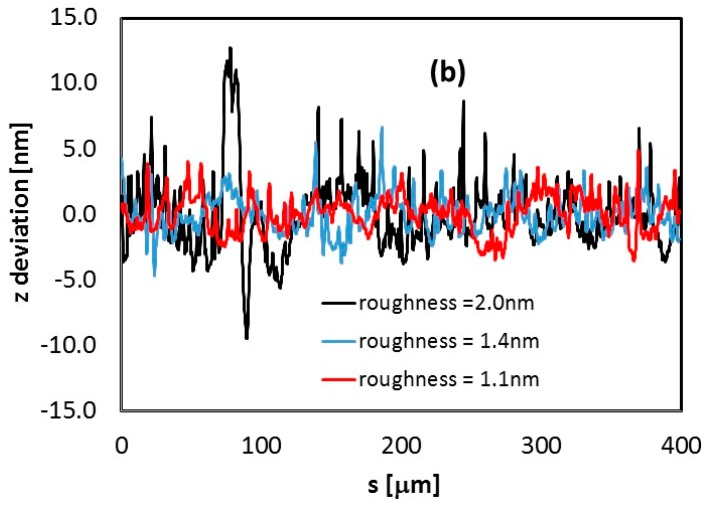
(**a**) Thickness profile (z direction) measured along a 900 μm path from the center of the In_2_O_3_ sample deposited on glass. (**b**) Thickness deviation from flat-film condition, estimated along three different profiles, 400 μm-long, within the same In_2_O_3_ 230 nm-thick film deposited on a glass substrate. For each of them, roughness is calculated as the average value of absolute deviation data.

Profiles of the sample were measured to determine the thickness of the sample (Figure 4a). Roughness of the In_2_O_3_ film deposited on glass has been also inspected by performing different scans with a profilometer on 400 μm length paths. Roughness data for three measurements on three different positions of the film are shown in Figure 4b. A roughness of 1–2 nm was measured, showing that LPED deposition grows compact dense films closely adhering on the substrate.

**Figure 5:**
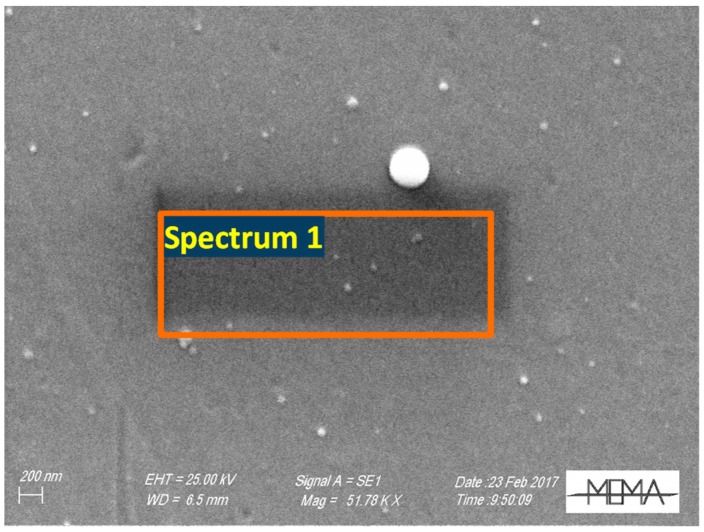
SEM micrography of a 230 nm thick film grown on glass.

SEM analyses performed with a Zeiss EVO MA15 Microscope support the profilometer measurements. As shown in Figure 5, the film appears dense and compact. Finally, microanalysis, performed in a volume with approximately 1 μm depth, with an Eds Oxford Inca 250, (see Table 1), confirms the presence of In_2_O_3_ in proper stoichiometry over the supporting glass window. 

### 2.2. Sensor Preparation and Characterization

**Figure 6:**
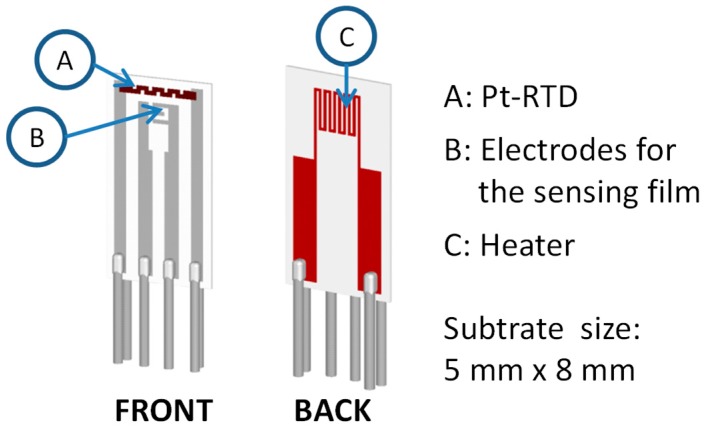
Alumina substrate.

Thin films were deposited by LPED on alumina substrates with the structure described in [18], and shown in Figure 6. The substrates are equipped with screen printed heaters on the backside, and with screen printed Pt based resistive temperature detectors (RTD, (a) in Figure 6) on the front. By means of these devices, the temperature of the tested film can be set and controlled by a feedback with an accuracy of few degrees in the range 120–400 °C [18]. The alumina used for the substrate (96% alumina as-fired, grain ~2 µm, surface roughness R_a_ ~800 nm) has large roughness: this choice grants a large surface area/volume ratio, which is fundamental for gas sensing applications. 

**Figure 7:**
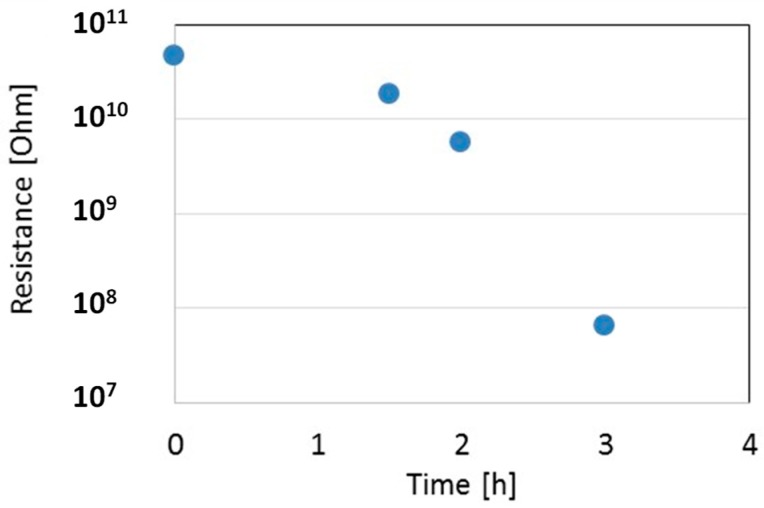
Resistance of the thin film as measured during deposition at 200 °C. Film growth rate is about 0.01 nm/s.

The LPED system was opportunely modified in view to allow for the remote control in real-time of the film resistance, sensor temperature, and heater supply. The temperature sensor, heater, and gas-sensor electrodes have been connected to set and monitor directly the substrate temperature, and to measure the In_2_O_3_ film resistance at different steps during deposition, with the aim of selecting a proper resistance value at 200 °C in the range 100 kΩ–10 MΩ. Figure 7 shows the measured resistance as a function of deposition time for one In_2_O_3_ film on an alumina substrate. During deposition, the temperature of the film, measured in real-time, was in the range 40–60 °C. To measure the resistance at 200 °C, the deposition was stopped for a few minutes, and the temperature of the film increased by means of the internal heater of the gas-sensor. Figure 7 shows a sudden decrease of resistance from almost infinite to a measurable value after about a 2 h deposition, when a first thin layer is settling through electrodes. Afterwards, the resistance decreases not linearly during deposition: this indicates that the substrate is not completely covered by the dense film, due to the high surface roughness of the substrate, so during the process both the surface area and the thickness grow together.

**Figure 8:**
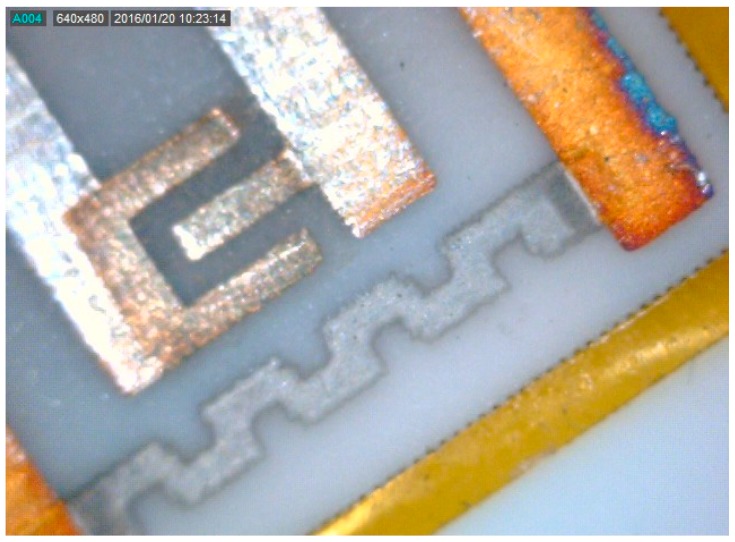
200× magnified photograph of the thin In_2_O_3_ film as deposited between electrodes of the gas-sensor device.

**Figure 9:**
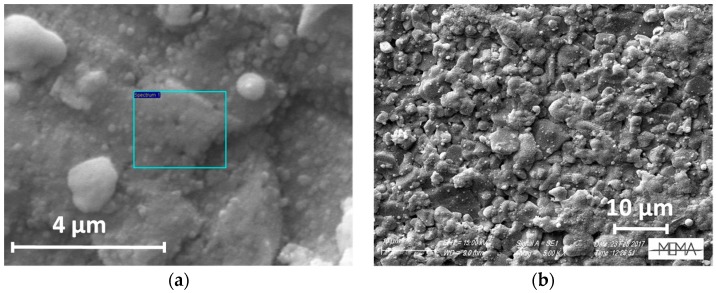
SEM images of a thin In_2_O_3_ film as deposited on the gas-sensor device with the LPED technique (**a**,**b**: different magnifications).

A sensor obtained with this technique is shown in Figure 8. Figure 9a,b show SEM micrographs of the sensing layer grown between the electrodes. The film covers the alumina substrate uniformly to mimic its high roughness, maintaining its large surface effective area.

Like many other metal oxides used for gas sensing, In_2_O_3_ is a large band-gap (*E_g_* = 3.55–3.75 eV) semiconductor that, irrespective of the preparation method, behaves as a doped n-type semiconductor due to favored bulk native point defects (oxygen vacancy donors), with an energy level in the band gap at less than 0.1 eV from the bottom of the conduction band.

Sensor characterization was performed by the measurement system described in [18], which allows for the accurate setting of the chemical environment in a test chamber both in terms of gas composition and flow (up to 500 mL/min), of humidity, and, finally, of film temperature (from 120 °C to 400 °C). In particular, a bench of digitally controlled flow-meters is used to properly mix the gases coming from certified gas tanks in order to obtain the desired concentrations of the target gases. The proposed sensors were tested in mixtures of dry air and CO or NO_2_. The experimental data were obtained in a constant flow of 200 mL/min. The results in the next section were obtained by evaluating the sensor response to a target gas as:

(2) $$RESPONSE=\frac{R-R_{0}}{R_{0}}\;\times \;100$$

where *R*_0_ indicates the baseline resistance in the carrier gas at the selected temperature, whereas R indicates the resistance of the film after a fixed time of exposure to the target mixture (4 min for all the reported experimental results).

## 3. Experimental Results

**Figure 10:**
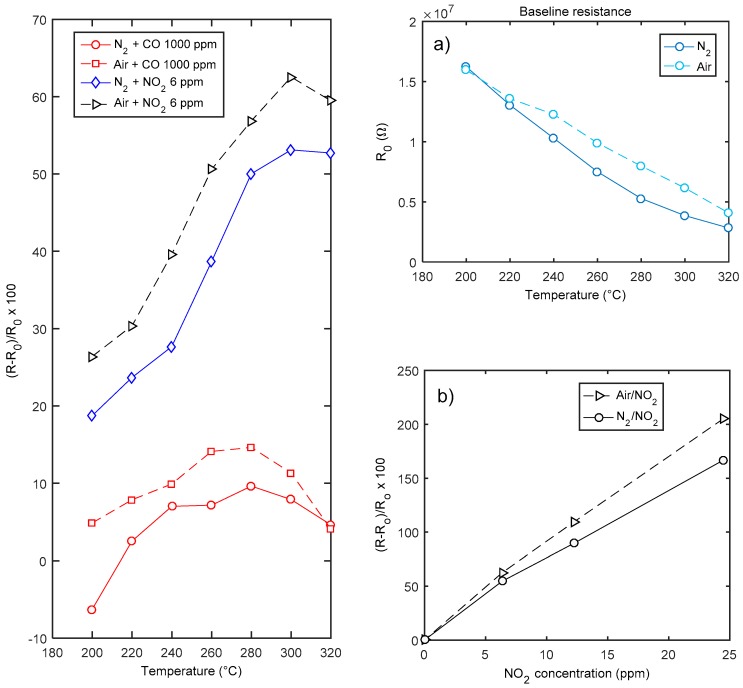
Gas sensing properties of a In_2_O_3_ sensor (deposition time 4 h, named hereafter sensor A). Leftmost plot—response as a function of temperature to CO and NO_2_. Rightmost plots: (**a**) baseline resistance in air as a function of temperature; (**b**) response to NO_2_ as a function of NO_2_ concentration at 300 °C.

The characterization results for sensors obtained after a deposition of 4 h (thickness around 150 nm) are summarized in Figure 10, where the response to CO and NO_2_ are shown. Moreover, in the figure the baseline resistance values in the two carrier gases are plotted as a function of temperature. As expected, the material behaves as an n-type semiconductor, and responds with an increased resistance to the oxidizing gases. The behavior in air and nitrogen is similar: oxygen seems to have little influence on the response to NO_2_ at high temperatures. Due to the material properties discussed in the introduction, the sensor shows a satisfactory response to NO_2_ (60% @ 6 ppm NO_2_ @ 320 °C in dry air). The response to NO_2_ in the tested concentration interval is fairly linear, both in nitrogen and in air. This behavior will be justified later by the proposed model. On the other hand, the sensor exhibits a negligible response to CO, which shows an anomalous behavior as acceptor-like extrinsic surface state (see Figure 10). This behavior, for the same temperature interval and in case of nano-grained thin films (thickness < 200 nm), was already described and discussed in [8].

In detail, it was found that CO always behaves as an acceptor like extrinsic surface state in N_2_ carrier gas, whereas in oxygen a reducing behavior is superimposed to the oxidizing behavior but only at high temperature. In [19], the acceptor-like behavior of CO was explained through the presence of humidity and oxygen in the environment. The experimental results seem only partially in agreement with this hypothesis, in fact using the dry inert gas carrier this anomalous behavior is still observable. 

The low sensitivity to CO and the small variation of the baseline resistance (see Figure 10) can be both explained by the absence of the depleted region at the surface. Nevertheless, also a small reactivity of the oxygen deficient surface with CO can be hypothesized. This last possible explanation is supported by measurements performed with a quartz crystal microbalance (AT-cut 10 MHz) at room temperature on whose gold electrodes a nano-layer (<100 nm thickness) of In_2_O_3_ was deposited with the same technique [20]. This sensor has a very little response to CO (no measureable response up to 2000 ppm CO), whereas it senses 12 ppm NO_2_.

**Figure 11:**
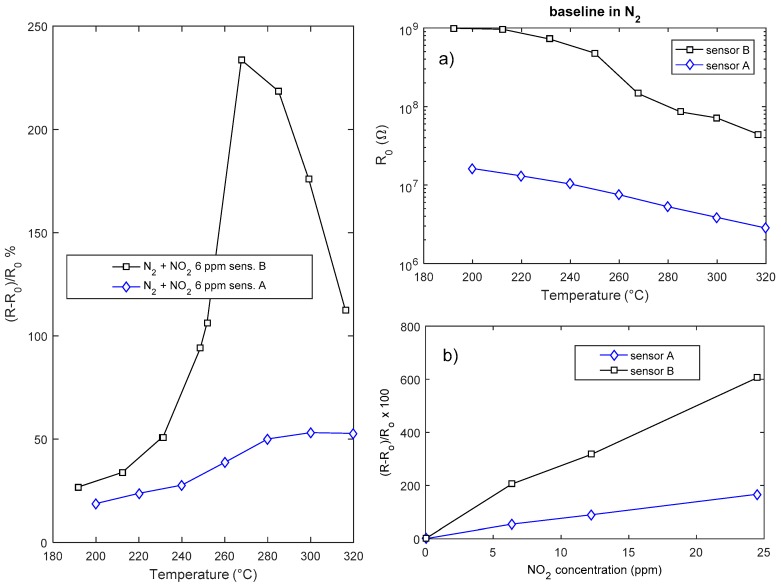
Gas sensing properties of two In_2_O_3_ sensors (sensor A-deposition time 4 h, sensor B-deposition time 2 h). Leftmost plot: response as a function of temperature to NO_2_. Rightmost plots: (**a**) Baseline resistance in air as a function of temperature. (**b**) Response to NO_2_ as a function of NO_2_ concentration at 300 °C.

The response of the sensors is highly dependent on the deposition time, i.e., on the layer geometry and film aspect ratio (Figure 11). Both the response and the speed of the sensor are beneficiated by a reduction of the sensor thickness; the drawback of reducing the deposition time is the reduction of the surface area and the very large value of the resistance that is obtained with deposition times shorter than 2 h.

**Figure 12:**
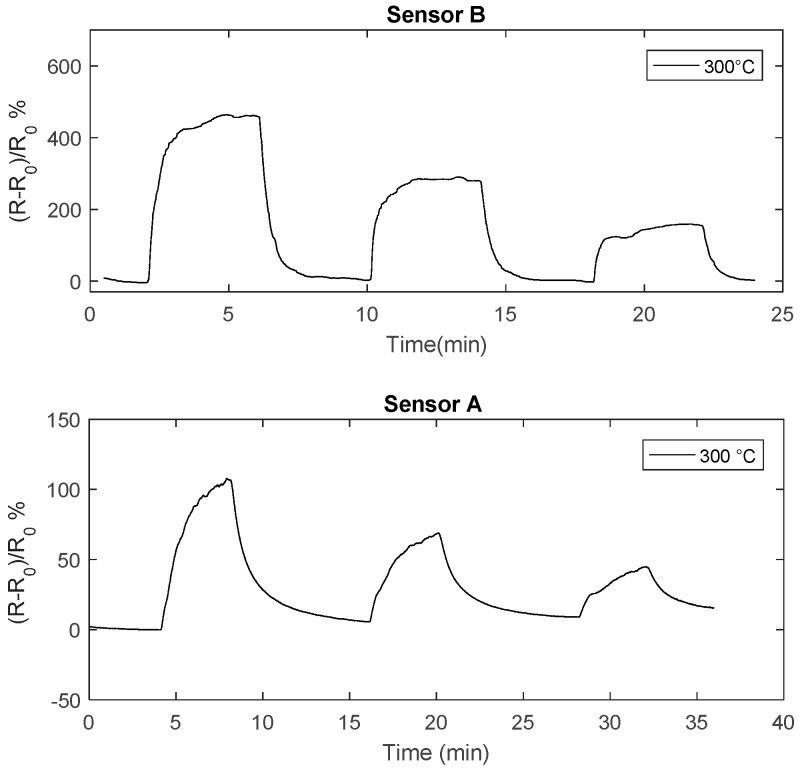
Resistance of the chemical film as a function of time when pulses (4 min length) with different concentration of NO_2_ are injected into the measurement chamber (24 ppm,12 ppm, 6 ppm). The total flow is 200 mL/min, carrier gas is N_2_. The NO_2_ concentrations are shown in the figure.

In Figure 12 the transient responses to NO_2_ of two sensors obtained with different deposition times are compared: it can be seen that reducing the deposition from 4 h time to 2 h (thickness about 70 nm), the response speed is highly improved, in fact the thinner sensor (sensor B) reaches the steady state and completely recovers in 4 min at temperatures larger than 290 °C.

## 4. Sensor Model for Oxidizing Gas

In general, in semiconductor metal oxides, gas sensing is mainly performed through three possible mechanisms affecting the electrical resistance (*R*):

(a) Change of free charge carrier concentration (*n*), due to capture/release at adsorbed species on surface, *R* ∝ 1/n.

(b) Change of potential barrier height (*qV_s_*) at the crystal domain (or grain) boundary, due to the presence of ionized adsorbed species on the crystallites surface. Barrier is due to charge carriers trapping at surface by acceptor surface states. The density of acceptor ionized surface states is determined by the population of intrinsic surface defects and by the possible presence of chemisorbed molecules. This gives rise to an exponential dependence of the electrical resistance on the barrier height, which is approximately related to the square of the trapped charge surface density: *R* ∝ *exp*(*qV_s_/kT*). The barrier height depends also on native acceptor surface defects (for n-type semiconductors) and on the concentration of the surrounding gas (oxidizing gas favoring the barrier, reducing gases likely suppressing it). On the contrary, if on the surface there are donor-like defects, a carrier enriched surface layer exists and the dependence of *R* on the charge surface density is no more exponential [20,21].

(c) Change of bulk electronic properties, such as the donor concentration (*N_d_*) and bulk charge carriers mobility (*μ_n_*), in this latter case *R* ∝ 1/(*N_d_ μ_n_*).

The first two mechanisms are pure surface phenomena driven by the adsorption/desorption of the surrounding gas and by the ionization/neutralization of adsorbed species. Obviously, diffusion toward the intergrain/boundary regions can also play a role in the kinetics.

The third process is a red/ox of the lattice, and takes into account possible diffusion of charged species in the bulk. According to [5], in In_2_O_3_ this last phenomenon seems to be negligible at temperatures lower than 350 °C.

Usually the most relevant phenomenon causing an exponential dependence of the resistance on the surface density of chemisorbed molecules is the (b) one. Compared with other well-known metal oxides (such as SnO_2_), In_2_O_3_ has a more complicated behavior as a gas sensor, due to the fact that the surface oxygen stoichiometry (oxygen deficiency) is such that the native surface defects may have a donor character. For this reason, it is possible that in reducing gases the surface is oxidized and no depleted region is formed at the surface of crystallites or grains, (on the contrary, there is a carrier enriched surface even in the presence of air [21,22], whereas in oxidizing environments a depleted region appears and the sensor behavior is the usual barrier height dependent one (b) [23]).

**Figure 13:**
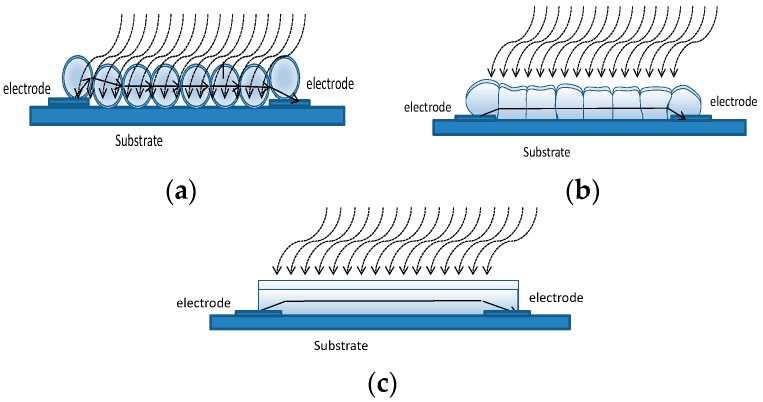
Sensor structure and model. (**a**) Porous layer. (**b**) Dense layer. (**c**) Compact layer.

It is known, that when the grain/film size goes below a certain critical value the influence of the grain/film geometry becomes important, and strongly influences the sensor response. In some previous works, the case of a thick film porous film made up of nano-spheres was treated by the authors and applied both to p-type and n-type materials [10,24]. The same model can be used also for thin films when they consist of loosely bound grains (porous layer) as shown in Figure 13a. This model could be used also for the thin nano-film described in this paper when short deposition times are used. Nevertheless, to ensure a reasonable value for the film resistance, as discussed in the previous section, the deposition has to be longer than 1.5 h. For long deposition time the film can become ‘dense’ (see Figure 13b). Assuming that no intrinsic acceptor like surface defect can be found at the grain boundary, such that no potential barrier exists at the grain interface, a possible model for the film is the compact thin-layer shown in Figure 13c. Moreover, Rombach in [23] showed that the vertical domain boundaries in the textured films have no detectable influence on the sensing behavior. In this case, the variation of resistance can be ascribed to the reduction of the current path cross section area, related to the creation of a depleted region on the surface when an oxidizing gas is present.

Let us consider a thin film of homogeneous material exposed to a mixture of air and NO_2_ (oxidizing gas); at the surface the two following chemisorption reactions are expected.

(3a) $$2S_{O}+O_{2}+2e^{-}\Leftrightarrow 2{\left(S_{O}-O\right)}^{-}$$

(3b) $$S_{NO_{2}}+NO_{2}+e^{-}\Leftrightarrow {\left(S_{O}-NO_{2}\right)}^{-}$$

where (*X-S_x_*)^−^ indicates an adsorbed and negatively ionized molecule of the species *X* at the adsorption site *S_x_*, and *e*^−^ indicates a free electron at the surface.

As a whole, the amount of charge localized at the surface is due both to intrinsic ionized defects and to the chemisorbed molecules with a total surface charge density −*qN_s_*, being *q* the electron charge and *N_s_* the total negatively charged species density that can be written as [1,25,26,27]

(4) $$N_{S}=\pm N_{i}+[{\left(S_{O}-O\right)}^{-}]+\left[{\left(S_{NO_{2}}-NO_{2}\right)}^{-}\right]$$

where *N_i_* indicates the density of ionized intrinsic defects that can be either acceptors or donors (actually, the surface of semiconductors tends always to be depleted [10]), and [*X*] indicates the surface density of the species *X*. In this work, we consider to have only extrinsic acceptor surface states due to the adsorption of both atomic oxygen and of the target gas NO_2_, a net negative charge, so that *N_s_* cannot usually assume a negative sign.

**Figure 14:**
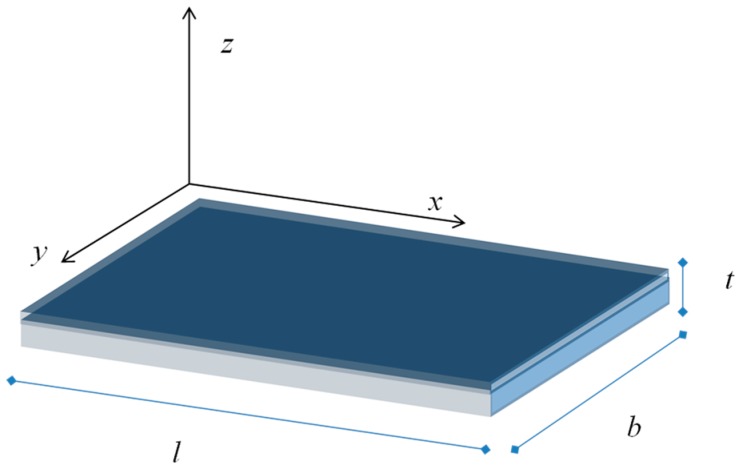
Film geometry.

Due to the trapped surface charge we expect that, when the equilibrium is reached, an electric field establishes in the grain to counterbalance the diffusion of free carriers which reach the surface from the bulk. We take into account an n-type semiconductor where the bulk free electron density is *N_d_* and the hole density is negligible. We consider a depleted surface—i.e., to have a net negative charge trapped at the surface—so that −*qN_s_*, the charge density, is negative and uniform on the surface. The positive charge density, *ρ,* in the film at the thermal equilibrium can be found by balancing the diffusion current related to the charge density gradient and the free electron drift current caused by the electric field. With reference to the geometry shown in Figure 14, being *t* << *b* and *t* << *l*, considering a homogeneous thin film and a uniform charge density at the surface, all the quantities can be considered to depend only on the of the z coordinate. Hence, the charge density can be found from the equation

(5) $$J_{tot}\left(z\right)=\sigma_{n}\left(z\right)E_{z}\left(z\right)-qD_{n}\frac{\partial n\left(z\right)}{\partial z}=0$$

where *J_tot_*(*z*) is the current density, *E_z_*(*z*) the electrical field, *σ_n_* = *qn*(*z*)*μ_n_* is the conductivity (*μ_n_* is the free electron mobility), *D_n_* is the diffusion coefficient of free electrons (*D_n_* = *μ_n_* *kT*/*q*), and *n*(*z*) is the free electrons density in the bulk.

Usually, the positive charge density in the depleted layer, *ρ*(*z*), is considered to be constant and equal to *qN_d_* (full depletion) so that in this layer no free electrons exist. Instead, we consider a spatial distribution of the charge that can be related to the free carrier density in this layer as

(6) $$n\left(z\right)=N_{d}-\frac{\rho \left(z\right)}{q}$$

From Equation (6) the conductivity in the depleted layer can be written as

(7) $$\sigma_{n}\left(z\right)=n\left(z\right)\mu_{n}q=\mu_{n}q\left(N_{d}-\frac{\rho \left(z\right)}{q}\right)$$

moreover

(8) $$E_{z}\left(z\right)\varepsilon =\int_{0}^{z}\rho \left(u\right)du$$

where *ε* is the electric permittivity.

Replacing the expressions in Equations (6)–(8) in Equation (5) we obtain the following integral-differential equation

(9) $$\mu_{n}\left(qN_{d}-\rho \left(z\right)\right)\int_{0}^{z}\rho \left(u\right)du=\varepsilon D_{n}\frac{\partial \rho \left(z\right)}{\partial z}$$

where 0 ≤ *ρ*(*z*) ≤ *qN_d_* holds, and *ρ*(0) assumes a value granting the following electro-neutrality condition

(10) $$\int_{0}^{t}\rho \left(u\right)du=qN_{s}$$

Equation (9) can be rewritten as

(11) $$\rho \left(z\right)+\frac{\varepsilon D_{n}}{\mu_{n}\int_{0}^{z}\rho \left(u\right)du}\frac{\partial \rho \left(z\right)}{\partial z}=qN_{d}$$

By numerically solving Equation (11) the charge density, the electrical field and the voltage distribution in the layer can be found.

It could be noted that since *ρ*(*z*) is always an increasing function of *z*, its local slope is determined by the length

(12) $$\lambda \left(z\right)=\frac{\varepsilon D_{n}}{\mu_{n}\int_{0}^{z}\rho \left(u\right)du}\geq \frac{k\varepsilon T}{q^{2}N_{d}t}$$

Therefore, it can be shown (see other works of the authors, e.g., [10,24]) that if the thickness of the layer is not much larger than the minimum value of *λ* given by the rightmost term of the inequality in Equation (12), *ρ*(*z*) slowly varies along z and its behavior is very far from the ‘fully depleted layer approximation’. It is evident that this condition is exactly equivalent to the usual condition

(13) $$t\gg \lambda_{D}$$

with $\lambda_{D}=\sqrt{\frac{k\varepsilon T}{q^{2}N_{d}}}$ being the Debye length.

When an external electrical field *E_ext_* is applied along the axis x, and considering it so small as not to interfere with the equilibrium due to the chemisorption, the following relationship holds for the drift current *J_x_*(*z*)

(14) $$E_{ext}=\sigma_{n}\left(z\right)J_{x}\left(z\right)$$

Hence the resistance of the layer can be found evaluating the following integral

(15) $$\frac{1}{R}=G=\frac{b}{l}\int_{0}^{t}\left(qN_{d}-\rho \left(u\right)\right)\mu_{n}du.$$

Actually, being the electro-neutrality condition (Equation (10)) always true, this integral is simply equal to

(16) $$G=\frac{b}{l}q\mu_{n}\left(N_{d}t-N_{s}\right)$$

Hence, if *G* = *G*(*N_s_*) is the conductance of the film in presence of the target gas, whereas *G*_0_ = *G*(*N*_*s*0_) is the conductance of the film in a reference condition, for instance in the carrier gas, we have

(17) $$\frac{G-G_{0}}{G_{0}}=\frac{N_{s0}-N_{s}}{N_{d}t-N_{s0}},$$

and, for the sensor response as defined in Equation (2)

(18) $$\frac{R-R_{0}}{R_{0}}=\frac{G_{0}-G}{G}=\frac{N_{s}-N_{s0}}{N_{d}t-N_{s}}$$

So for the dense compact film a linear relationship between the charged species density and the conductance is expected. It can be seen that the sensor response depends on the film thickness and on the bulk carrier density. The smaller are these quantities the larger the sensor response. So, very thin pure films would provide, in principle, very good sensitivities. Obviously, both thickness and bulk carrier density cannot be pushed to low values limitless because they determine the film resistance value. A lower limit for both is set by the maximum measureable resistance and by the desired measurement range. In particular, very large resistances lead to heavy problems in measurement system design, and limit the measurement accuracy. Moreover, a low *N_d_* value and a small thickness can bring to the depletion of the entire film and to response saturation.

**Figure 15:**
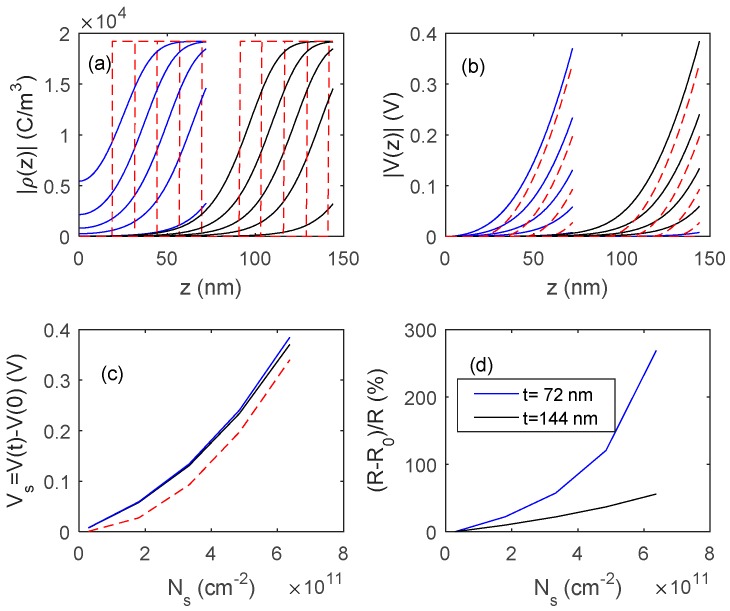
Distribution of the charge density ρ (plot (**a**)), and of the voltage (plot (**b**)) inside the film as a function of z for different values of charged surface species density, *N_s_*, and for two films thickness, 72 nm and 144 nm. *N_s_* in the range 0.1 × 10^11^ cm^−2^–6.3 × 10^11^ cm^−2^. (**c**) Surface voltage barrier as a function of the density of charged surface species. Solid lines: proposed model, dashed lines: fully depleted model. (**d**) Sensor responses to the variation of surface charge density. Data for In_2_O_3_ are taken from [28,29] *N_d_* = 1.2 × 10^17^ cm^−3^.

**Figure 16:**
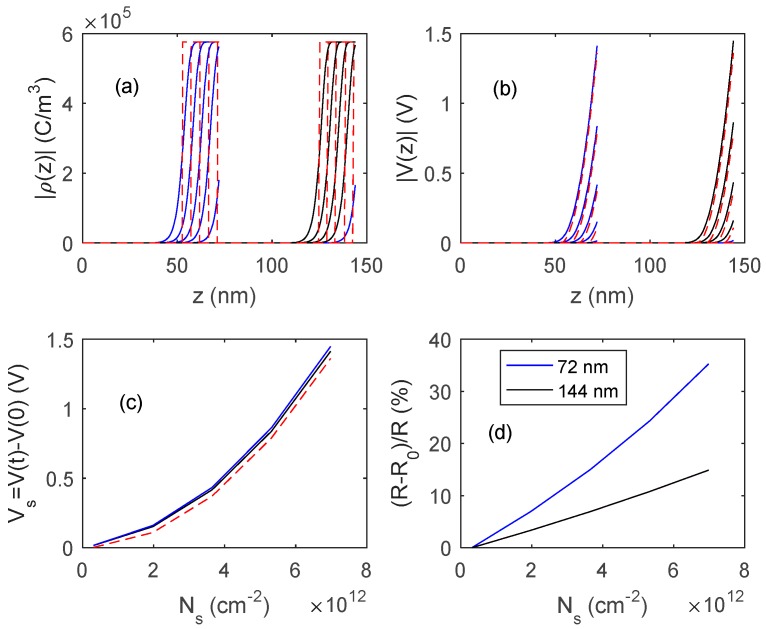
Distribution of the charge density, ρ (plot (**a**)) and of the voltage (plot (**b**)) inside the film as a function of z for different values of charged surface species density, *N_s_*, for two films thickness, 72 nm and 144 nm. *N_s_* in the range (0.3 × 10^12^ cm^−2^–7.2 × 10^12^ cm^−2^). (**c**) Surface voltage barrier as a function of the density of charged surface species. Solid lines: proposed model, dashed lines: fully depleted model. (**d**) Sensor responses to variation of surface charge density. Data for In_2_O_3_ are taken from [28,29] *N_d_* = 3.6 × 10^18^ cm^−3^.

In Figure 15 and Figure 16, the outputs of the proposed model, obtained by numerically solving Equation (11), are shown, and compared with those obtained with fully depleted model. The presented results concern two different In_2_O_3_ materials, the first one (Figure 15) is a pure material with a very low bulk carrier concentration (*N_d_* = 1.2 × 10^17^ cm^−3^) whereas the second one (Figure 16) is characterized by a much larger *N_d_* value (3.6 × 10^18^ cm^−3^). In simulations, *N_s_*_0_ is assumed to be negligible, so the response is simply $\left(R-R_{0}\right)/R_{0}=N_{s}/\left(N_{d}t-N_{s}\right)$. A comparison of the simulated results with the experimental data indicates that the obtained films behave similarly to those shown in Figure 15, where the reduction of the film thickness from 144 nm to 72 nm provides a very large gain in terms of response, indicating that *N_d_t* is close to *N_s_*. 

## 5. Conclusions

In this paper, the feasibility of gas sensors based an In_2_O_3_ nano-layer deposited on alumina printed circuit substrates by means of low temperature pulsed electron deposition (LPED) technique was explored. As a prime novelty, we have implemented our deposition system with a remote control of the film electrical resistance directly during the growth process. This work shows a first proof of principle of this specific new feature which, providing a more sophisticated tool to tailor in situ the ultimate sensitivity of gas-sensors, opens the way to a new generation of devices with potentially increased functionality. The sensing gas properties of the developed devices were assessed, through experiments and exploiting a model taking into account the nano-structure of the sensing layer. It was shown that the sensors have a linear response in a large range of gas concentration (chemisorbed molecules density), a good sensitivity to NO_x_ and very low sensitivity to CO. In detail, as far as sensitivity to NO_2_ is concerned, the optimum temperature range is 280–300 °C. Moreover, the lower the film thickness, the higher the sensitivity and the speed (but the higher also the film resistance): at 300 °C the response to NO_2_ increases by a factor of ~3, in the range 0 ppm–25 ppm, if films of 70 nm are used instead of 150 nm (and the baseline resistance increases as well between 1 and 2 orders of magnitude). The stability of the sensors was at present verified only by preliminary encouraging tests. A more exhaustive measurement campaign to characterize both stability and durability is ongoing.

## Figures and Tables

**Table 1:** Micronalysis data concerning the average value of the region enclosed by the red box area with a depth of approximately 1 µm (partly in glass).

**Spectrum 1**	**Weight% O**	**Weight% Si**	**Weight% Ca**	**Weight% In**	**Weight% Total**
	31.20	20.66	2.27	45.87	100
	**Atomic%** **O**	**Atomic%** **Si**	**Atomic%** **Ca**	**Atomic%** **In**	
	62.07	23.41	1.80	12.72	
